# ﻿Novel genomic resources contribute to the systematics of threatened arboreal deer mice of the genus *Habromys* Hooper & Musser, 1964 (Cricetidae, Neotominae) within a neotomine–peromyscine phylogeny

**DOI:** 10.3897/zookeys.1179.108759

**Published:** 2023-09-11

**Authors:** Susette Castañeda-Rico, Lillian D. Parker, Evelyn Sánchez, Sheccid Rivas-Trasvina, Melissa T. R. Hawkins, Cody W. Edwards, Jesús E. Maldonado

**Affiliations:** 1 Smithsonian-Mason School of Conservation, Front Royal, VA, USA Smithsonian-Mason School of Conservation Front Royal United States of America; 2 Center for Conservation Genomics, Smithsonian National Zoo and Conservation Biology Institute, Washington, DC, USA Center for Conservation Genomics, Smithsonian National Zoo and Conservation Biology Institute Washington United States of America; 3 Department of Biology, George Mason University, Fairfax, VA, USA George Mason University Fairfax United States of America; 4 Department of Anthropology, University of Oklahoma, Norman, OK, USA University of Oklahoma Norman United States of America; 5 Department of Vertebrate Zoology, Division of Mammals, National Museum of Natural History, Washington DC, USA Department of Vertebrate Zoology, Division of Mammals, National Museum of Natural History Washington United States of America

**Keywords:** Conservation, *Habromyslophurus*, Mesoamerica, mitogenomes, museomics, phylogenomics, ultraconserved elements

## Abstract

The Crested-tailed deer mouse, *Habromyslophurus*, is one of seven arboreal species within the genus *Habromys*. Species of this genus are monotypic, relatively rare, and occur in low densities. Their geographical distribution is highly fragmented due to being restricted to montane cloud forest in Mesoamerica and they are of conservation concern. All *Habromys* species are endemic to Mexico, except *H.lophurus*, which is also distributed in Guatemala and El Salvador. In this study, we obtained and characterized the first mitogenome and several thousand nuclear ultraconserved elements (UCEs) of *H.lophurus* to determine its phylogenetic position within neotomine–peromyscine mice. Its mitogenome sequence (16,509 bp) is only the second complete mitogenome obtained for this poorly known genus. We also obtained the first nuclear genomic data for *H.lophurus*, including 3,654 UCE loci, as well as a partial mitogenome of *H.simulatus* (6,349 bp), and 2,186 UCE for the outgroup *Holochilussciureus*. Phylogenetic analyses that included our newly generated genomic data coupled with previously published data from other neotomine–peromyscine mice confirm the placement of *H.lophurus*, *H.simulatus*, and *H.ixtlani* within a highly supported clade. The *Habromys* clade was nested within a clade that also contains members of the genus *Peromyscus* and provides further support for the hypothesis of the paraphyly of *Peromyscus*. These genomic resources will contribute to future phylogenomic studies that aim to further elucidate the evolutionary history of this rare and critically endangered genus of rodents.

## ﻿Introduction

The genus *Habromys* Hooper & Musser, 1964, considered a relict, comprises seven monotypic species (*H.simulatus* (Osgood, 1904), *H.chinanteco* (Robertson & Musser, 1976), *H.delicatulus* Carleton, Sánchez & Urbano-Vidales, 2002, *H.schmidlyi* Romo-Vázquez, León-Paniagua & Sánchez, 2005, *H.lophurus* (Osgood, 1904), *H.lepturus* (Merriam, 1898), and *H.ixtlani* (Goodwin, 1964)) which inhabit montane regions of Mesoamerica, ranging from central-southern Mexico to north-eastern El Salvador (Hooper 1968; Carleton et al. 2002; León-Paniagua et al. 2007; Rogers et al. 2007). Species of *Habromys* are arboreal and restricted to montane cloud forest and moist-oak forest. The montane cloud forest, a relict ecosystem, is one of the most highly endangered and fragmented habitats mainly because of land conversion for grazing and agriculture (Toledo-Aceves et al. 2011). The association between species of the genus *Habromys* and this ecosystem makes their geographical distribution highly discontinuous and susceptible to climate change and anthropogenic activities (Carleton et al. 2002; Contreras-Medina et al. 2022). These species are elusive and rare, with small population sizes, and are poorly represented in scientific collections (Romo-Vázquez et al. 2005; León-Paniagua et al. 2007; Castañeda-Rico et al. 2011). In addition, each species is currently found in only one or a few localities, resulting in micro-endemic distributions (León-Paniagua et al. 2007; Rogers et al. 2007; Castañeda-Rico et al. 2011). The characteristics mentioned above increase the risk of extinction for all species within this genus. Members of the genus *Habromys* are considered characteristic elements of the Neotropical montane cloud forest and contribute to the recognition of Mesoamerica as a biodiversity hotspot (Contreras-Medina et al. 2022). Despite the biological importance of this genus, little is known about its evolutionary history. León-Paniagua et al. (2007) and Rogers et al. (2007), using mitochondrial genes (*ND3*, *ND4L*, partial *ND4*, and *Cyt b*, respectively), conducted the only two molecular phylogenetic studies of the genus to date.

The crested-tailed deer mouse, *Habromyslophurus*, is distributed in Nuclear Central America, from Chiapas, Mexico to Guatemala and El Salvador (León-Paniagua et al. 2007; Rogers et al. 2007). It is possible that this species also occurs in small patches in western Honduras (Carleton et al. 2002; Contreras-Medina et al. 2022) but, to date, no specimens have been identified or captured in that region. Robertson and Musser (1976) and Carleton et al. (2002) found that the body size in populations from Chiapas is smaller than those in Guatemala and El Salvador, but they considered that this distinction did not warrant taxonomic recognition. However, León-Paniagua et al. (2007), using three mitochondrial genes, suggested that both *H.lophurus* and *H.simulatus* consist of two geographically isolated and restricted species; i.e. *H.lophurus* from the highlands of Chiapas, Mexico, and northern Guatemala, and *H.* sp. nov. 2 from Sierra Las Minas, eastern Guatemala to El Salvador, and *H.simulatus* from central Sierra Madre Oriental, Mexico and *H.* sp. nov. 1 from the Sierra Mazateca, Oaxaca, Mexico. In addition, Castañeda-Rico et al. (2011), using microsatellites, found that *H.simulatus* included two distinct evolutionary units. The objective of this study was to generate additional mitochondrial data representing the full mitochondrial genome, as well as the first nuclear genomic data ever generated for the species—i.e. thousands of ultraconserved element loci—to place *H.lophurus* within a neotomine–peromyscine phylogeny and to provide genomic resources for future phylogenomic studies with a denser taxon sampling.

## ﻿Materials and methods

### ﻿Specimen sampling and lab work

**Figure 1.**
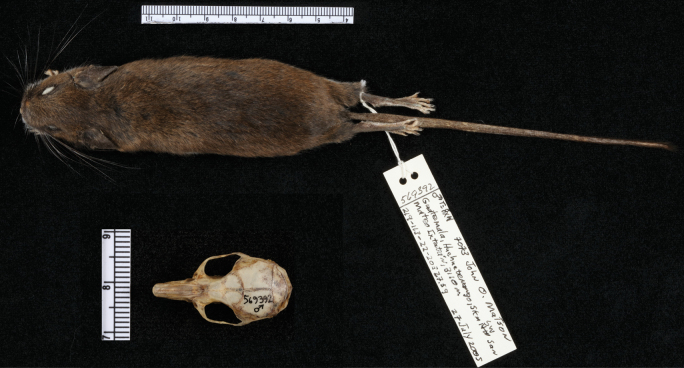
Voucher specimen of *Habromyslophurus* (USNM 569392) from which the mitogenome and ultraconserved elements were obtained in this study. This species may be identified by its crested tail and usually by the absence of a white underside of the tail. Currently, there are no pictures available of live individuals of this species. Photograph by Katie Sayers.

We used a tissue sample of *Habromyslophurus* associated with a male museum voucher specimen (Fig. 1), both of which are accessioned at the Smithsonian Institution’s National Museum of Natural History with catalog number USNM 569392, to sequence the first publicly available mitogenome and several thousand nuclear ultraconserved elements (UCEs) of this species. The specimen was collected in 2005 in Guatemala, Huehuetenango, 5 km southwest of San Mateo Ixtatán (15°48′36.36″N, 91°30′19.44″W).

DNA was extracted from the tissue sample (ca 1.5 mm^2^ of frozen internal organ) using the DNeasy Blood and Tissue Kit (QIAGEN, Inc., Valencia, CA, USA), following the manufacturer’s protocol, and quantified using a Qubit (Thermo Fisher, Waltham, MA, USA) fluorometer with a 1× dsDNA HS assay kit. DNA was sheared to an average length of 250 base pairs (bp) using a Bioruptor Pico sonicator (Diagenode Inc., Denville, NJ, USA) with a pulse of 30 s on, 30 s off for 90 cycles. A dual-indexed library with TruSeq-style indices (Meyer and Kircher 2010), amplified with Kapa HiFi HotStart Ready Mix (Roche Sequencing), was prepared using the Kapa HyperPrep kit (Roche Sequencing, Indianapolis, IN, USA) with ½ reactions following the manufacturer’s protocol. The myBaits Mito kit for the house mouse (*Musmusculus* Linnaeus, 1758) and the myBaits UCE Tetrapods 5Kv1 kit, both produced by Daicel Arbor Biosciences, were used for target-enrichment to capture mitogenomes and UCEs, respectively. The post-enrichment libraries were amplified with 14 cycles of PCR using universal Illumina primers and Kapa HiFi Hotstart Ready Mix, and sequenced on a NovaSeq 6000 (Illumina, Inc., San Diego, CA, US) at the Oklahoma Medical Research Foundation, Oklahoma City (combined with samples from other projects). We also reanalyzed raw data published by Castañeda-Rico et al. (2020, 2022a), under BioProjects PRJNA606805 and PRJNA818347, to obtain a mitogenome of *Habromyssimulatus* and UCEs of *Holochilussciureus* Wagner, 1842.

### ﻿Bioinformatics and phylogenetic analyses

For mitogenomes, read quality analysis of the FASTQ format files was conducted using FastQC v0.11.5 (Andrews 2010). Low-quality reads and adapter contamination were removed using TrimGalore v0.6.5 (https://github.com/FelixKrueger/TrimGalore). Prinseq-lite v0.20.4 (Schmieder and Edwards 2011) was used to remove exact duplicates (-derep1,4). Cleaned reads were mapped to a reference (*Habromysixtlani* GenBank KY707304) using the Geneious algorithm in Geneious Prime 2021.2.2 (https://www.geneious.com) with default parameters (Medium-Low sensitivity, Maximum mismatches = 20%, Maximum gaps = 10%). Consensus sequences were generated with Geneious, using 5X as the lowest coverage to call a base. The MAFFT v7.45 plug-in (Katoh and Standley 2013) in Geneious was used to align the consensus sequences and references. We transferred annotations from the reference mitogenome and translated all protein-coding genes (PCGs) to rule out the presence of nuclear copies of mitochondrial genes (NUMTs) and to check for frame shifts or stop codons.

For the UCEs, raw data were processed following the PHYLUCE v1.6.7 pipeline (Faircloth 2016). Illumiprocessor v2.10 (Faircloth 2013) and TrimGalore v0.6.5 (https://github.com/FelixKrueger/TrimGalore) were used to trim adapters, barcode regions and low-quality bases. Trinity v2.8.5 (Grabherr et al. 2011) was used to assemble trimmed reads into contigs. Contigs matching the UCE 5K probes set were aligned using MAFFT v7.4 (Katoh and Standley 2013; Nakamura et al. 2018) and edge trimming was performed. The resulting alignment was filtered based on matrix completeness (missing data). A 90% matrix (10% of taxa missing for each locus) was used for the phylogenetic analyses. Informative sites were quantified with the PHYLUCE script phyluce_align_get_informative_sites.py.

To determine the phylogenetic position of *H.lophurus*, a Bayesian Inference (BI) analysis was performed using MrBayes v3.2.6 (Ronquist et al. 2012) under the best model and partition scheme (by gene and codon position – 6 partitions) selected by PartitionFinder v2.1.1 (Lanfear et al. 2016) for the mitogenome dataset and without partitions for the UCE dataset. We included previously published Neotominae and Sigmodontinae mitogenomes and UCEs downloaded from GenBank; KY707304 and KY707303 (Sullivan et al. 2017), MT078816–MT078819 PRJNA606805 (Castañeda-Rico et al. 2020), OL685394 PRJNA818347 (Castañeda-Rico et al. 2022a), ON528108–ON528119 PRJNA838631 (Castañeda-Rico et al. 2022b), and OP432689 PRJNA880321 (Castañeda-Rico et al. 2023). The final datasets included 21 and 20 species for mitogenomes and UCE, respectively (Table 1). *Holochilussciureus* was used as an outgroup for both datasets. The BI analyses ran for 50 million generations sampling every 1,000 generations with a burn-in of 25%. Output parameters were visualized using Tracer v1.7.1 (Rambaut et al. 2018) to check for convergence between runs. FigTree v1.4.4 (http://tree.bio.ed.ac.uk/software/figtree/) was used to visualize the phylogenetic trees. All of these analyses were performed on the Smithsonian Institution High Performance Computing Cluster (Smithsonian Institution: https://doi.org/10.25572/SIHPC).

**Table 1.** Specimens examined in this study using mitogenomes and UCEs with species name, accession number collection/ID study, GenBank accession number, and GenBank BioProject. Smithsonian Institution’s National Museum of Natural History (USNM), Museum of Texas Tech University (TTU/TK), Museo de Zoología “Alfonso L. Herrera” Facultad de Ciencias UNAM (MZFC), University of Michigan Museum of Zoology (UMMZ), and Museum of Vertebrate Zoology, University of California, Berkeley (MVZ).

Species	Number Scientific Collection/ID	Mitogenome (GenBank Number)	UCE (GenBank BioProject)
*Habromyslophurus*	USNM569392/USNM569392	OP936043	PRJNA907399
*Habromyssimulatus*	MZFC10104/HBR031	Figshare	PRJNA606805
*Habromysixtlani*	– /TK93158	KY707304	–
*Peromyscushooperi*	USNM340233/USNM340233	OP432689	PRJNA880321
*Peromyscusmegalops*	USNM340233/USNM340233	ON528115	PRJNA838631
*Peromyscusattwateri*	TTU143738/TK185663	ON528112	PRJNA838631
*Peromyscusaztecus*	USNM569848/USNM569848	ON528113	PRJNA838631
*Peromyscuspolionotus*	USNM585473/USNM585473	ON528117	PRJNA838631
*Peromyscuscrinitus*	TTU146966/TK193714	ON528114	PRJNA838631
*Peromyscusmekisturus*	UMMZ88967/UMMZ88967	MT078818	PRJNA606805
*Peromyscusmelanophrys*	MZFC3907/MQ1229	MT078816	PRJNA606805
*Peromyscusperfulvus*	– /MCP119	MT078817	PRJNA606805
*Peromyscuspectoralis*	MZFC10465/FCR176	MT078819	PRJNA606805
*Peromyscusmexicanus*	MZFC11150/MRM030	–	PRJNA606805
	TTU82723/TK93144	KY707303	–
*Podomysfloridanus*	TTU97866/TK92501	ON528118	PRJNA838631
*Neotomodonalstoni*	TTU82668/TK93098	ON528110	PRJNA838631
*Onychomysleucogaster*	TTU146304/TK171574	ON528111	PRJNA838631
*Reithrodontomysmexicanus*	TTU138428/TK178510	ON528119	PRJNA838631
*Isthmomyspirrensis*	USNM565924/USNM565924	ON528108	PRJNA838631
*Neotomamexicana*	TTU104969/TK150189	ON528109	PRJNA838631
*Holochilussciureus*	MVZ190356/MVZ190356	OL685394	PRJNA818347

## ﻿Results and discussion

**Figure 2.**
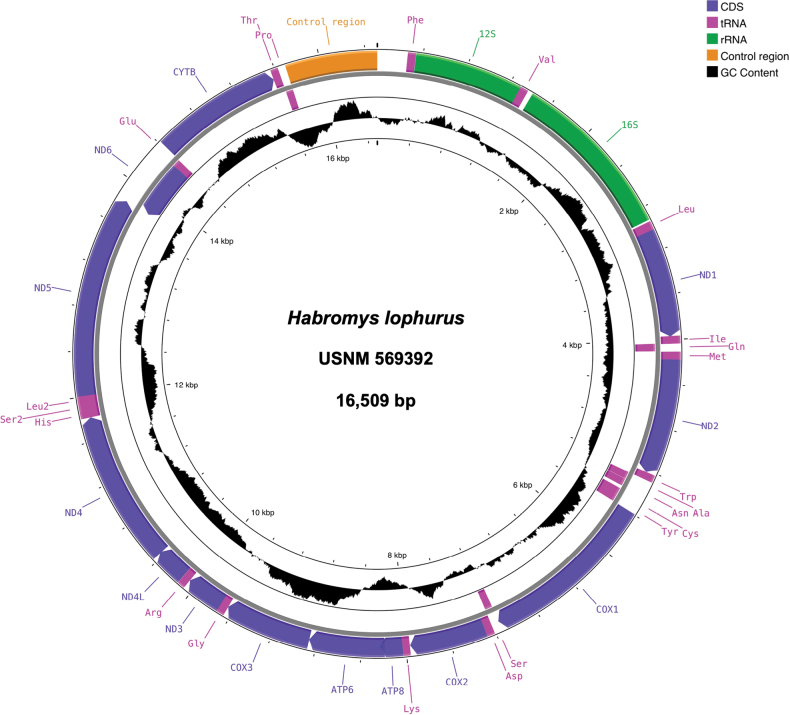
Mitogenome map of the crested-tailed deer mouse *Habromyslophurus* (USNM 569392, GenBank accession number OP936043) depicting the gene organization. The inner circle shows the CG content along the mitogenome.

The assembled complete mitogenome sequence of *Habromyslophurus* is 16,509 bp long (GenBank accession number OP936043), which covers 100% of the reference genome sequence. The average coverage per base was 2,701X with 347,009 unique sequence reads mapped to the reference genome. The sequence contained the standard features present in a vertebrate mitochondrial genome including one non-coding control region, 2 rRNA genes (*12S rRNA* and *16S rRNA*), 22 tRNA genes, and 13 PCGs including NADH dehydrogenase (*ND1*, *ND2*, *ND3*, *ND4*, *ND4L*, *ND5* and *ND6*), cytochrome *c* oxidase (*COX1*, *COX2*, and *COX3*), ATP synthase (*ATP6* and *ATP8*) and cytochrome *b*. The base composition was 34.4% A, 23.8% C, 12.7% G, 29.1% T and the GC content was 36.5%. The mitogenome map (Fig. 2) was visualized using CGView tool (Stothard and Wishart 2005) in the online resource Proksee (https://proksee.ca/). We also obtained a partial mitogenome (6,349 bp) of *H.simulatus* (https://doi.org/10.25573/data.23563929). The assembled mitogenome, including missing data, is 16,062 bp long with average coverage per base of 4.2X and 515 unique reads mapped to the reference. The *H.simulatus* mitogenome includes the same features (one control region, 2 rRNA genes, 22 tRNA genes, and 13 PCGs) as *H.lophurus* mitogenome. The base composition was 35.6% A, 23.8% C, 12.8% G, 27.7% T, and the GC content was 36.7%.

The UCE processing recovered 209,371 and 556,910 contigs for *H.lophurus* and *Holochilussciureus* after Trinity assembly, respectively. A total of 3,654 UCE loci were obtained for *H.lophurus* and 2,186 for *H.sciureus*. The incomplete matrix contained 4,415 UCE loci (*N* = 20; average = 3,145; min = 1,087; max = 3,859). The 90% matrix included 438 UCE loci with an average of 15 informative sites and 649 bp length per locus.

**Figure 3.**
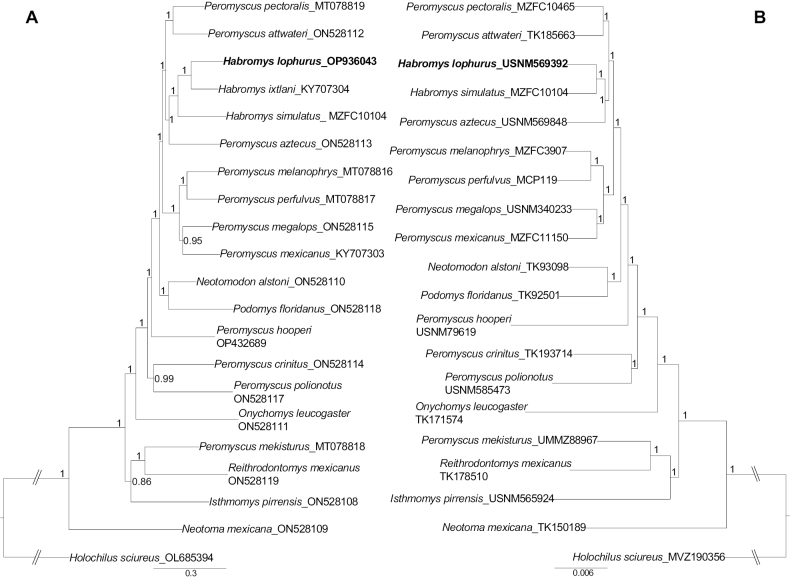
Bayesian phylogenetic tree of neotomine–peromyscine mice using (**A**) complete mitogenomes and (**B**) ultraconserved elements (UCEs). Nodal support is provided with posterior probabilities values. GenBank accession numbers follow species names for the mitogenome-based phylogeny and the accession number collection for the UCE-based phylogeny.

Our phylogenetic analyses support the placement of *H.lophurus*, *H.simulatus*, and *H.ixtlani* within a clade with high nodal support (Fig. 3). However, the clade comprising the genus *Habromys* is nested within a clade that contains members of the genus *Peromyscus* (sensu stricto; Carleton 1980). Therefore, our data support the hypothesis that *Peromyscus* is paraphyletic (Bradley et al. 2007; Miller and Engstrom 2008; Platt et al. 2015; Sullivan et al. 2017; Castañeda-Rico et al. 2022b). In addition, we corroborated previous results by Castañeda-Rico et al. (2022b, 2023) where members of the genus *Habromys* are sisters to *P.aztecus*, a member of the *Peromyscusaztecus* species group, and this clade is sister to a clade comprising members of the *Peromyscustruei* species group (*P.attwateri* and *P.pectoralis*). The difference between those previous studies and ours is that they only included *H.simulatus* in the UCE-based phylogeny and *H.ixtlani* in the mitogenome-based phylogeny (one sample for each dataset). In contrast, in this study, we have expanded our sampling and have included two species (*H.simulatus* and *H.lophurus*) in the UCE-based phylogeny and three species (*H.simulatus*, *H.ixtlani*, and *H.lophurus*) in the mitogenome phylogeny. Another difference among these studies is that Castañeda-Rico et al. (2022b, 2023) did not test for the monophyly of the genus; however, these additional three species enabled us to confirm the monophyly of *Habromys*. The sister relationship between the genus *Habromys* and *P.aztecus* coincides with Osgood (1909), who recognized a close affinity between *H.lophurus* + *H.lepturus* + *H.simulatus* and forms of the *Peromyscusaztecus* species group. In contrast, León-Paniagua et al. (2007) and Rogers et al. (2007) did not resolve the relationship of *Habromys* with any of the outgroups used in their studies due to low nodal support values. Rogers et al. (2007) attributed the poor resolution of the outgroups due to the practical limitations of the high saturation rate of the *Cyt b* gene with increased phylogenetic depth. In this sense, we showed that our mitochondrial and nuclear genomic datasets were able to help overcome this issue.

Within *Habromys*, the mitogenome-based phylogeny showed that *H.lophurus* is sister to *H.ixtlani*, and both are sisters to *H.simulatus*. This relationship was not supported by the UCE-based phylogeny due to a lack of UCE data for *H.ixtlani*. A close relationship between *H.lophurus* and *H.ixtlani* is supported by previous morphological studies that suggest *Habromys* includes two main lineages: one containing the smallest and more arboreal species (*H.simulatus*, *H.schmidlyi*, *H.delicatulus*, and *H.chinanteco*) and the other including the largest and less arboreal species (*H.ixtlani*, *H.lepturus*, and *H.lophurus*) (Carleton et al. 2002). Based on our limited sampling of species (3 out of 7) within *Habromys*, we cannot resolve the relationships within the genus or test the phyletic affinity among morphologically similar species. However, we show that using mitogenomes and nuclear genome-wide data provides well-resolved phylogenies that can be used to resolve or support opposite phylogenetic hypotheses. For example, León-Paniagua et al. (2007), using only three mitochondrial genes, found that *H.lophurus* is sister to *H.ixtlani* + (*H.lepturus* + *H.chinanteco*), followed by a sister relationship to *H.delicatulus* + *H.schmidlyi*, where *H.simulatus* is the most divergent species within the genus; however, Rogers et al. (2007), using one mitochondrial gene, found that *H.lophurus* is sister to a clade including *H.ixtlani* + *H.lepturus*, and this clade is sister to *H.delicatulus*, where *H.simulatus* + *H.delicatulus* are the most divergent of the genus, but they did not include *H.schmidlyi* in their study. Neither of these studies, based on limited data from mitochondrial genes, found support for the two clades suggested by Carleton et al. (2002), and they did not recover the same topology. In addition, some of the clades in those genetic studies were not highly supported. We expect that the mitogenomes and UCE loci generated in this study will contribute to future phylogenomic studies that aim to further elucidate the phylogenetic relationships and the evolutionary history of this critically endangered genus.

All *Habromys* species are of conservation concern given the increasing annual deforestation rates for montane cloud forest (Rogers et al. 2007; Contreras-Medina et al. 2022) as well as other characteristics described above. The International Union for Conservation of Nature (IUCN) established that populations of all *Habromys* are decreasing but only *H.simulatus*, *H.ixtlani*, *H.schmidlyi*, *H.chinanteco*, and *H.lepturus* are considered Critically Endangered (Álvarez-Castañeda et al. 2018a, 2018b, 2018c, 2018d; Vázquez 2018), while *H.delicatulus* is considered Endangered (Vázquez 2017) and *H.lophurus* Near-Threatened (Emmons and Vázquez 2019). However, only *H.simulatus* is listed in the Mexican Official Norm NOM-059-SEMARNAT-2010 (Secretaría de Medio Ambiente y Recursos Naturales 2010) under the category of Special Protection (Pr). The NOM-059 is the official document generated by the Mexican government that includes threatened animal and plant species.

Efforts to determine the correct status of these species have been stifled because of the very limited number of studies that have focused on this genus. Furthermore, a recent study looking at distributional patterns and conservation of the genus *Habromys* (Contreras-Medina et al. 2022) proposed that all *Habromys* species except *H.lophurus* must be included in the NOM-059 because their geographical distribution covers less than 5% of the Mexican territory based on the known distribution and their species-distribution models. However, they did not consider the particular case of *H.lophurus* and *H.simulatus*, where it has been proposed that they consist of two geographically isolated species (León-Paniagua et al. 2007). This urgently needs to be resolved in future studies and, if it is confirmed, it would provide additional support for the protection of these two geographically restricted and imperiled species as it would change the status of *H.lophurus* to an Endangered or Critically Endangered species. We propose that all species in this genus should be listed in the Mexican Official Norm under the category of Threatened (A) or Critically Endangered (P) and as Critically Endangered (CR) by the IUCN.

## ﻿Additional information

### Author ORCIDs

Susette Castañeda-Rico https://orcid.org/0000-0002-4301-3579

Lillian D. Parker https://orcid.org/0000-0003-3370-9473

Melissa T. R. Hawkins https://orcid.org/0000-0001-8929-1593

Cody W. Edwards https://orcid.org/0000-0001-5912-6693

Jesús E. Maldonado https://orcid.org/0000-0002-4282-1072

### Data availability

The mitochondrial genome sequences are available in GenBank at https://www.ncbi.nlm.nih.gov/genbank/ under the accession number OP936043 and Figshare (https://doi.org/10.25573/data.23563929). The associated BioProject, BioSample and SRA numbers are PRJNA907399, SAMN31979917, and SRR22493489, respectively.
